# *Valsa mali* Pathogenic Effector VmPxE1 Contributes to Full Virulence and Interacts With the Host Peroxidase MdAPX1 as a Potential Target

**DOI:** 10.3389/fmicb.2018.00821

**Published:** 2018-04-25

**Authors:** Mian Zhang, Hao Feng, Yuhuan Zhao, Linlin Song, Chen Gao, Xiangming Xu, Lili Huang

**Affiliations:** ^1^State Key Laboratory of Crop Stress Biology for Arid Areas, College of Plant Protection, Northwest A&F University, Yangling, China; ^2^NIAB East Malling Research, East Malling, United Kingdom

**Keywords:** Apple *Valsa* canker, effector protein, cell death suppressor, virulence factor, peroxidase

## Abstract

The *Valsa* canker, caused by *Valsa mali* (*V. mali*), is a destructive disease of apple in Eastern Asia. Effector proteins are important for fungal pathogenicity. We studied a candidate effector VmPxE1 isolated based on the genome information of *V. mali.* By using the yeast invertase secretion assay system, VmPxE1 was shown to contain a signal peptide with secretory functions. VmPxE1 can suppress BCL-2-associated X protein (BAX)-induced cell death with a high efficacy of 92% in *Nicotiana benthamiana*. The expression of *VmPxE1* was upregulated during the early infection stage and deletion of *VmPxE1* led to significant reductions in virulence on both apple twigs and leaves. VmPxE1 was also shown to target an apple ascorbate peroxidase (MdAPX1) by the yeast two-hybrid screening, bimolecular fluorescence complementation and *in vivo* co-immunoprecipitation. Sequence phylogenetic analysis suggested that MdAPX1 was an ascorbate peroxidase belonging to a subgroup of heme-dependent peroxidases of the plant superfamily. The ectopic expression of *MdAPX1* in the mutant of *VmPxE1* significantly enhanced resistance to H_2_O_2_, while the presence of *VmPxE1* seems to disturb *MdAPX1* function. The present results provide insights into the functions of VmPxE1 as a candidate effector of *V. mali* in causing apple canker.

## Introduction

Secreted by bacteria, oomycetes, and fungi, effectors are defined as small rich cysteine secreted proteins, contributing to the pathogen virulence (Vleeshouwers and Oliver, 2014). Pathogen and host have been co-evolving, resulting in the establishment of multi-layered pathogen offense and host defense systems. Pathogen-associated molecular patterns (PAMPs) are recognized by pattern recognition receptors (PRRs), and then PAMP-triggered immunity (PTI) of the host is induced (Boller and He, 2009; Albert, 2013). Under the pressure from host defense, pathogens secrete effectors to suppress host defense, leading to effector-triggered susceptibility (ETS). ETS results in production of plant resistance proteins (R proteins) and leads to the second layer of immune response, the effector-triggered immunity (ETI), leading to heavy oxidative burst and hypersensitive reaction (HR) of the host (Jones and Dangl, 2006; Dodds and Rathjen, 2010).

Filamentous pathogenic effectors have been reported to interfere with several aspects of host immunity (Rovenich et al., 2014). Some act as inhibitors of proteases, such as Pit2 from *Ustilago maydis* and EPI10 from *Phytophthora infestans* (Tian et al., 2005; Mueller et al., 2013). Some influence enzymes related to the ROS pathway. For example, two cytoplasmic effectors of *Phytophthora sojae* interact with catalases to regulate H_2_O_2_ concentration (Zhang et al., 2015). An *U. maydis* effector Pep1 targets a maize peroxidase POX12 *in vivo* and suppresses the early immune responses of maize (Hemetsberger et al., 2012). Some effectors may competitively bind defense-related proteins to disturb the host-recognition system, such as PsXLP1, from *P. sojae*, acting as a decoy to shield the true virulence factor (Ma et al., 2017), and Ecp6 from *Cladosporium fulvum*, targeting chitin to suppress the chitin-induced immunity (de Jonge et al., 2010). Moreover, the resistance signal pathway could also be disturbed by pathogenic effectors. For instance, the effector HaRxL44 from *Hyaloperonospora arabidopsidis* breaks down the SA-triggered immunity (Caillaud et al., 2013) and the *U. maydis* effector Cmu1 interdicts SA biosynthesis (Djamei et al., 2011). Thus, pathogenic fungi have effectors that could function via several mechanisms to defeat/avoid host defense systems.

*Valsa mali* is a necrotrophic fungus belonging to Ascomycete and causes *Valsa* canker on apple, a destructive disease of apple in the Eastern Asia. In China, this disease resulted in significant economic losses (Lee et al., 2006; Wang et al., 2011). Previous research identified 193 candidate effector proteins (CEPs) with unknown functions and predicted 779 secreted proteins of *V. mali* with rich cysteine residues (average length of 233 amino acids) (Yin et al., 2015). The ability to suppress BAX triggered PCD is an important initial criterion for screening pathogenic effectors (Wang et al., 2011a). BAX is a member of the Bcl-2 family proteins, triggering cell death when expressed in plants. The cell death-promoting function of BAX in plants correlated with the upregulated expression of the defense-related protein PR1, which strongly suggests that BAX activates an endogenous cell-death program, one specific host defense reaction (Lacomme and Santa Cruz, 1999). Using this initial screening system for BAX responses, eight effectors have been identified and one of them (VmEP1) shown to be an important virulence factor of *V. mali* (Li et al., 2015). There are probably other *V. mali* effectors yet to be identified (Ke et al., 2014; Yin et al., 2015). Further research is needed to identify other effectors and study their functions.

In this study, we identified VmPxE1 as a cell death suppressor, contributing to the full virulence of *V. mali* and directly targeting a peroxidase of apple tree.

## Materials and Methods

### Strains and Culture Conditions

*Valsa mali* strain 03-8 was cultured at 25°C in the dark on potato dextrose agar (PDA) medium. *Nicotiana benthamiana* plants were maintained at 25°C with a daily 13 h:11 h light:dark regime. *Agrobacterium tumefaciens* strain GV3101 used for agro-infiltration experiments was cultured on Luria-Bertani medium at 28°C. *Escherichia coli* strain JM109 used for storing and propagating plasmids was cultured on Luria-Bertani medium at 37°C. All the strains are stored in the Laboratory of Integrated Management of Plant Diseases at the College of Plant Protection, Northwest A&F University, Yangling, China.

### Construction of *V. mali* cDNA Libraries

Total RNA was extracted using the RNeasy Micro kit (Qiangen, Shenzhen, China) according to the manufacturer’s protocol from (a) *V. mali* mycelia grown on PDA medium for 3 days, and (b) apple twig tissues of *Malus domestica* borkh. cv. ‘Fuji’ inoculated with *V. mali* mycelium plugs [0 h (i.e. immediately before inoculation), 6, 12, 18, 24, 36, 48, 72 h post inoculation (hpi)]. First-strand cDNA was synthesized by the RevertAidTM First Strand cDNA Synthesis Kit (Fermentas, Shenzhen, China) according to the manufacturer’s protocol.

### Plasmid Constructs

Targeted genes were amplified from the cDNA library using the Ex-taq mix DNA polymerase (Takara, Dalian, China). For *A. tumefaciens* infiltration assays in *N. benthamiana*, PCR products were cloned into the corresponding vectors (Giraldo and Valent, 2013) with the restriction enzyme digestion and T4 DNA ligase (Takara, Dalian, China). Primers used for plasmid constructs are listed in **Supplementary Table S1**. Sequences of all plasmids were confirmed by Sangon Biotech, Shanghai.

### Sequence Analyses

In our previous study, the whole genome shotgun sequences of *V. mali* were deposited at DDBJ/EMBL/GenBank under the accession JUIY01000000. The secretome and CEPs of *V. mali* were predicted from materials obtained in previous studies (Li et al., 2015; Yin et al., 2015). Pfam^1^ was used to predict protein domain structure, and SignalP 4.1^2^ to signal peptides. An unrooted phylogenetic tree was constructed by MEGA5 with the neighbor-joining method.

### *Agrobacterium tumefaciens* Infiltration Assays

Agro-infiltration assays were carried out following the previously described procedure (Dou et al., 2008). Strain GV3101 carrying an expression plasmids was grown in LB medium containing kanamycin (50 μg/ml) for transient expression; cells were re-suspended in 10 mM MgCl2 (pH 5.6) with the suspension adjusted to an OD600 of 0.5. Bacteria were infiltrated with a syringe to the upper leaves of 5-week-old *N. benthamiana* plants. As a control, plants were infiltrated with bacteria carrying an empty pGR106 vector. Symptoms were assessed 4–5 days after infiltration. Each assay was performed three times.

### Secretory Function Validation of Putative N-Terminal Signal Peptide of VmPxE1

The yeast invertase secretion assay was performed to validate the function of the putative N-terminal signal peptide of VmPxE1. The predicted signal peptide of VmPxE1 was cloned to pSUC2 and transformed into yeast strain YTK12 with the lithium acetate method (Geitz et al., 1995). The CMD-W medium (0.08% tryptophan dropout supplement, 2.5% sucrose, 0.65% yeast N base without amino acids, 0.1% glucose, and 2% agar) was used to select YTK12 colonies with pSUC2 empty vector or pSUC2-VmPxE1sigp. For validating invertase secretion, positive colonies on the CMD-W medium were transferred to YPRAA plates (1% yeast extract, 2% raffinose, 2% peptone, and 2 mg/ml antimycin A) containing raffinose as the only carbohydrate source. The untransformed YTK12 colonies, YTK12 colonies transformed with an empty pSUC2 vector, and the non-secreted Mg87 protein from *Magnaporthe oryzae* were used as negative controls (Gu et al., 2011); the YTK12 colonies carrying the signal peptide of Avr1b from oomycete was used as a positive control (Shan et al., 2004; Gu et al., 2011).

### RNA Extraction and Transcript Level Analysis

*VmPxE1* transcript level was measured by qRT-PCR in apple twig tissues inoculated with *V. mali* strain 03-8 sampled at 0 (immediately before inoculation), 6, 12, 18, 24, 36, 48, and 72 hpi. Total RNA was extracted using the RNAeasy R Plant Mini Kit (Qiagen, Shenzhen, China) following the recommended protocol. First-strand cDNA was synthesized using a RT-PCR system (Promega, Madison, WI, United States) following the manufacturer’s instructions. SYBR green qRT-PCR assays were performed to quantify transcript levels; *G6PDH* of *V. mali* was chosen as a housekeeping gene (Yin et al., 2013). There were three biological replicates for each treatment. Primers used for qRT-PCR are given in **Supplementary Table S2**.

### Generation of *VmPxE1* Mutants

A reaction with three components using the *Neo* gene as a selective marker was performed for single gene deletion. The *Neo* gene was amplified with primers Neo-F and Neo-R from PFL2. The upstream and downstream flanking sequences of *VmPxE1* were amplified using primer pairs 1F/2R, 3F/4R, respectively. Then, deletion cassette for homologous recombination was generated by double-joint PCR as described previously (Yu et al., 2004). The primer pair CF/CR was used for nest-PCR and produced the gene-replacement construct. Protoplasts of *V. mali* were prepared and then the gene-replacement construct was transformed into the protoplasts as previously described (Gao et al., 2011). Each putative single gene deletion mutant was verified by PCR using four primer pairs (5F/6R, 7F/NeoR, NeoF/8R, and NeoF/NeoR) to detect the target gene, upstream-neo fusion segment, neo-downstream fusion segment, and the neo gene, respectively. Southern blot hybridization using the DIG DNA Labeling and Detection Kit II (Roche, Mannheim, Germany) was performed to confirm deletion mutants. All primers used for gene deletion are given in **Supplementary Table S1**.

### Complementation of the Deletion Mutants

The entire *VmPxE1* gene with upstream 2000 bp was amplified with primers pDL2-*VmPxE1*-F and pDL2-*VmPxE1*-R cloned into plasmid pDL2 with the yeast gap repair approach (Bruno et al., 2004; Zhou et al., 2012). The resulting construct, pDL2-*VmPxE1*, was transformed into protoplasts of the *VmPxE1* gene deletion mutants. Complementation was selected by geneticin (G418) and hygromycin, and confirmed by PCR using the primer pair 5F/6R.

### Pathogenicity, Conidiation, and Vegetative Growth of Mutants

For pathogenicity assay, detached apple (*M. domestica* borkh. cv. ‘Fuji’) twigs and leaves were inoculated with the deletion mutant Δ*VmPxE1*, complementation mutant Δ*VmPxE1/VmPxE1* and wild type following the method previously described (Oliva et al., 2010). The experiments were repeated twice with fifteen replicates in each repeat experiment. Vegetative growth and conidiation was examined at 3 and 40 days, respectively. Data were analyzed by Student’s *t*-test using the SAS software package (SAS Institute, Cary, NC, United States).

### Yeast Two-Hybrid Screening

The yeast two-hybrid system was performed for screening *VmPxE1* interacting proteins (Ito et al., 2001). The *VmPxE1* coding region was cloned into the bait vector pGBKT7 without the signal peptide sequence and pGBKT7-*VmPxE1* was transformed into yeast strain AH109. Yeast cells carrying pGBKT7-*VmPxE1* were transformed with cDNA library that was constructed into the prey vector pGADT7 using mRNA isolated from the junction of diseased (infected by *V. mali* wild type strain 03-8) and healthy twigs. Candidate clones growing on the SD/-Leu-Trp-His medium were picked to SD/-Leu-Trp-His+X-α-galactosidase medium for confirmation of the interaction.

### Bimolecular Fluorescence Complementation

A published BiFC procedure (Waadt et al., 2008) was used. *VmPxE1* and *MdAPX1* were cloned into the vector pSPYNE(R)173 and the vector pSPYCE(M), respectively. *A. tumefaciens* strain GV3101 carrying the expression vector pSPYNE(R)173-*VmPxE1* was co-injected with *A. tumefaciens* strain GV3101 carrying pSPYCE(M)-*MdAPX1* at the 1:1 ratio into 4-week-old *N. benthamiana* plants. About 48–60 h after co-agroinfection, live-cell imaging was taken with a two-photon confocal laser scanning microscopy (Olympus FV1000MPE) with the FV10-ASW 3.1 software suite. The assay was repeated twice.

### Protein Extraction and Western Blots

About 60–72 h after agro-infection, the treated *N. benthamiana* leaves were rapidly frozen in liquid nitrogen and triturated in mortar, then total proteins of the leaf tissues were extracted with Plant Total Protein Extraction Kit, following the manufacturer’s instruction (P0028, Beyotime technologies, Shanghai, China).

For the western blot analysis, total proteins from leaves were separated using SDS–PAGE (40V, 8 mA) and transferred onto nitrocellulose membranes (60V, 150 mA). The blot was blocked with Western Quick Block mixture (P0023B, Beyotime Technologies, Shanghai, China) for 1 h at ambient temperature and followed by (1) incubation with the monoclonal antibody (1:500) in WB primary antibody diluent (P0103, Beyotime technologies, Shanghai, China) for 2 h at ambient temperature; (2) being washed with TBS containing 0.1% Tween-20 (TBST), and (3) reacting with goat anti-mouse IgG horseradish peroxidase (HRP) (1:500) (A0216, Beyotime Technologies, Shanghai, China) for 1 h at ambient temperature. Finally, the nitrocellulose membrane was immunostained with 3,30-diaminobenzidine (DAB) for 10 min in the dark before visualization.

### *In Vivo* Co-immunoprecipitation

For the *in vivo* Co-IP assay, *A. tumefaciens* strain GV3101 carrying the expression vectors PICH86988-*HA*-*MdAPX1* and pBin-*GFP*-*VmPxE1* was co-agroinfiltrated into *N. benthamiana* leaves. Harvest proteins 500 μL was added to equilibrated GFP-trap beads (Chromotek) and fully mixed at 4°C overnight on a slow shake incubator, and then centrifuged with the supernatant discarded. Re-suspended GFP-trap beads were washed by pre-cold wash buffer twice and then transferred to 100 μL 2 × SDS loading buffer. The sample was boiled for 10 min to dissociate for immunoblot analysis using the anti-GFP or anti-HA antibody.

### Ectopic Expression of *MdAPX1* in *V. mali*

The entire *MdAPX1* gene sequence was cloned into plasmid pDL2 with the yeast gap repair approach (Bruno et al., 2004; Zhou et al., 2012). Then PEG-mediate protoplast transformation was performed to transferred pDL2-MdAPX1 into *V. mali* wild type 03-8 and the *VmPxE1* gene deletion mutant Δ*VmPxE1* with the hygromycin B phosphotransferase gene (hph) as a selective marker. Transformed colonies were selected by hygromycin B. Then the stress resistance against H_2_O_2_ was measured by culturing wild type 03-8, transformed colonies of *03-8/MdAPX1* and Δ*VmPxE1/ MdAPX1* on PDA with 0.06% H_2_O_2_ at 25°C. Fungal colony size was assessed 3 days after treatment. There were 30 petri dishes per treatment. The expression of *MdAPX1* was identified with RT-PCR. The experiment was repeated twice.

## Results

### VmPxE1 From *V. mali* Suppressed BAX-Induced Cell Death in *N. benthamiana*

**FIGURE 1:**
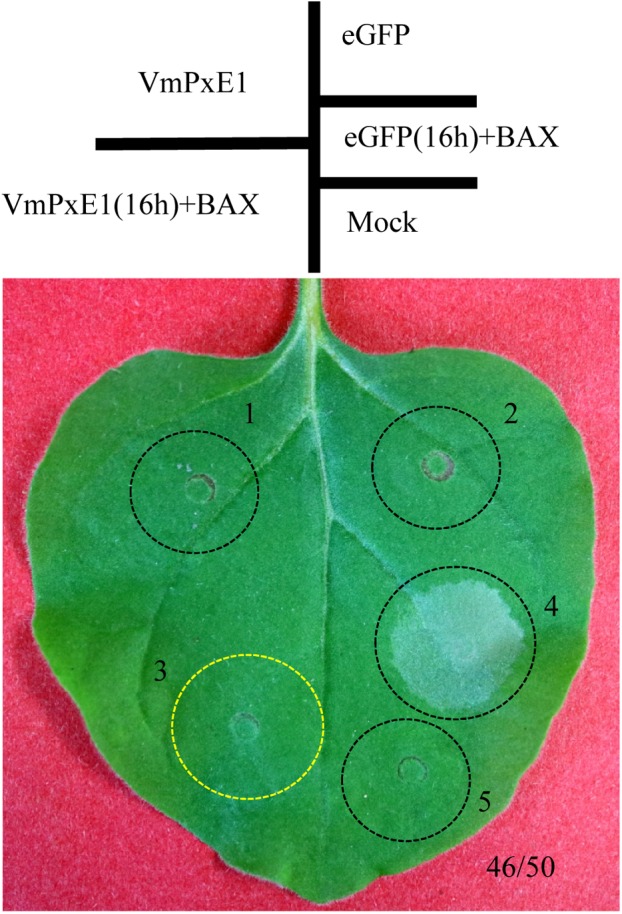
Transient expression of *VmPxE1* in *N. benthamiana* leaves by agro-infiltration. Symptoms on leaves of *N. benthamiana* were assessed 5 days after inoculation.

A protein of unknown functions, *VmPxE1* (KUI70334.1) residing on chromosome 6 of *V. mali*, was found to contain 213 amino acid residues with a predicted signal peptide. Transient expression assay in *N. benthamiana* leaves with *BAX* suggested that *VmPxE1* is an effective cell death suppressor effector with a cell death suppression ratio of 92% (46/50) (**Figure 1**). Subsequent RT-PCR and western blot with an anti-eGFP and anti-HA antibody confirmed that *VmPxE1*, *BAX*, and *eGFP* were all expressed in *N. benthamiana* (**Supplementary Figures S1A,B**).

### Secretory Function Validation of the Putative N-Terminal Signal Peptide of VmPxE1

**FIGURE 2:**
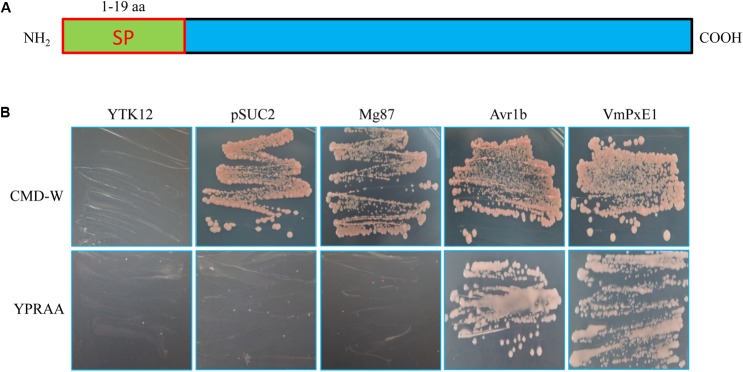
Secretory function validation of the putative N-terminal signal peptide of VmPxE1 using the yeast invertase secretion assay: **(A)** a schematic diagram of putative VmPxE1 signal peptide; **(B)** the sequence of the putative VmPxE1 signal peptide fused in-frame to the invertase sequence in the pSUC2 vector and then transformed into yeast strain YTK12. The empty pSUC2 and the non-secreted Mg87 protein from *Magnaporthe oryzae* were used as negative controls and the secreted effector Avr1b from oomycete as a positive control. Only the yeast strains that are able to secrete invertase can grow on both CMD-W and YPRAA media.

The *VmPxE1* amino acid sequence contained a predicted signal peptide at the position of 1-19 aa (**Figure 2A**). The invertase mutant yeast strain YTK12 containing VmPxE1 signal peptide recombinant plasmids could grow on YPRAA medium (with raffinose instead of sucrose) indicating that the invertase was secreted (**Figure 2B**). This result indicates that the putative N-terminal signal peptide of VmPxE1 was a functional secretion signal peptide.

### Transcription Level of Effector Gene *VmPxE1*

**FIGURE 3:**
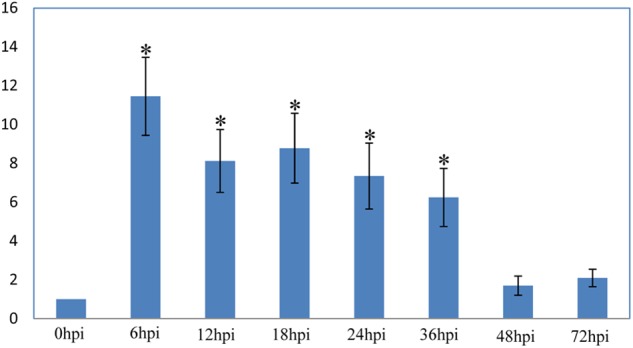
Relative expression level of *VmPxE1* at 0, 6, 12, 18, 24, 36, 48, and 72 h post inoculation of apple twigs and leaves with *G6PDH* as a housekeeping gene. Results were presented as a mean fold change in the expression relative to the expression at 0 h. Significant difference in pathogenicity was indicated with asterisks (*P* < 0.05). Each experiment was repeated twice and error bars indicate SEM.

*VmPxE1* expression was quantified at 0 (immediately before inoculation), 6, 12, 18, 24, 36, 48, and 72 hpi on apple twigs with *G6PDH* as a housekeeping gene. *VmPxE1* was upregulated for all sampling points. The expression of *VmPxE1* was increased significantly at 6, 12, 18, 24, and 36 hpi (**Figure 3**).

### The *VmPxE1* Is Not a Necessary Growth Factor but a Virulence Factor of *V. mali*

To estimate the contribution of *VmPxE1* to virulence, this gene was deleted (**Supplementary Figure S2A**). The deletion mutant Δ*VmPxE1* was obtained and further confirmed by PCR analysis (**Supplementary Figure S2C**) and Southern hybridization (**Supplementary Figure S2B**). The complementation mutant Δ*VmPxE1/VmPxE1* was generated and confirmed by PCR analysis (**Supplementary Figure S2D**).

**FIGURE 4:**
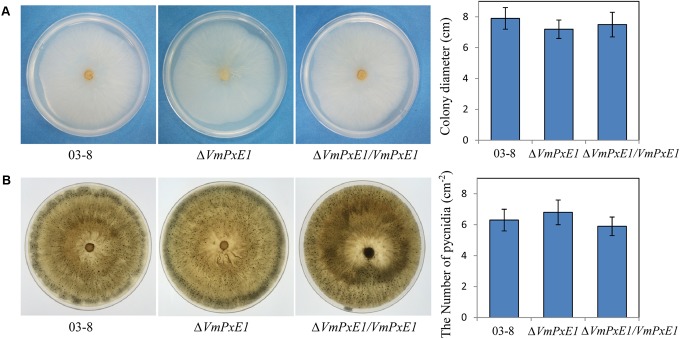
Vegetative growth and sporulation of *V. mali* wild type strain 03-8, Δ*VmPxE1* and Δ*VmPxE1/VmPxE1* on PDA for 3 days **(A)** or 40 days **(B)** at 25°C. Colony diameter was measured by crossing method and pycnidia were counted per square centimeter (cm^-2^). Bars indicate SD of the mean of 30 individual dishes.

**FIGURE 5:**
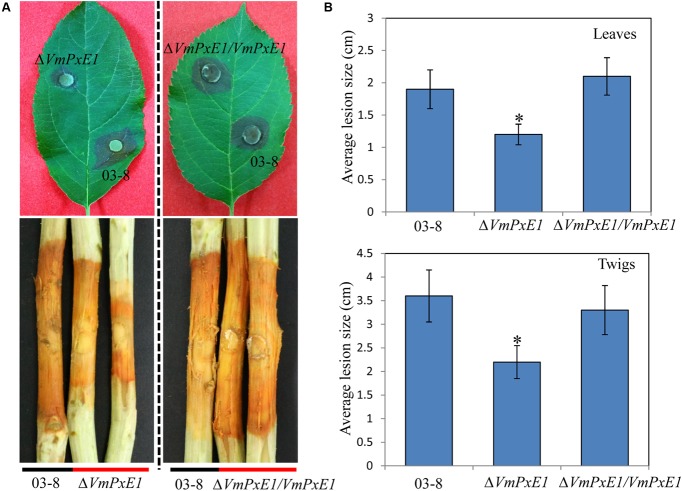
Pathogenicity of *V. Mali* wild type strain 03-8, deletion mutant Δ*VmPxE1* and Δ*VmPxE1* complementation mutant (Δ*VmPxE1/VmPxE1*) when inoculated onto leaves and twigs of *Malus domestica* borkh. cv. ‘Fuji.’ **(A)** Symptom on twigs and leaves after inoculation. **(B)** Average lesion size of scabs on twigs and leaves. Observation was made 60 and 120 h after inoculation for leaves and twigs, respectively. Asterisk indicates significant (*P* < 0.05) differences. Bars indicate SD of the mean of 30 individual host plants.

*In vitro* inoculation showed that deletion of *VmPxE1* did not affect vegetative growth (**Figure 4A**) and sporulation of *V. mali* (**Figure 4B**). However, Δ*VmPxE1* showed significant reduction in virulence on both apple twigs and leaves (**Figure 5A**), about 42.1 and 38.9% on leaves and twigs, respectively (**Figure 5B**). The complementation mutant Δ*VmPxE1/VmPxE1* had the similar level of infection as the wild type 03-8.

### VmPxE1 Interacted With MdAPX1, an Apple Ascorbate Peroxidase

**FIGURE 6:**
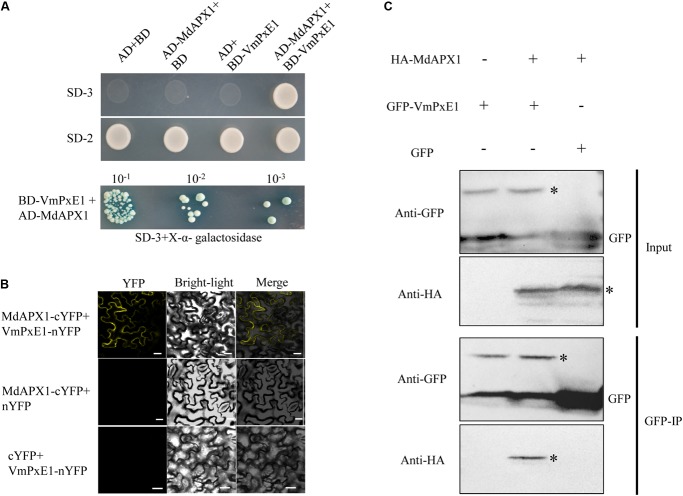
Interaction of VmPxE1 with MdAPX1. **(A)** Yeast cells were cultured on SD-Trp-Leu-His and SD-Trp-Leu as control. Positive yeast clones were cultured on SD-3+X-α-galactosidase with ladder concentration of yeast suspension (10^-1^, 10^-2^, and 10^-3^) for further confirmation. **(B)** Bimolecular fluorescence complementation showed that VmPxE1 interacted with MdAPX1 in leaf cells of *N. benthamiana*. VmPxE1–nYFP and MdAPX1–cYFP were co-expressed in *N. benthamiana* by agro-infiltration. The yellow fluorescence was observed 48–72 h post inoculation (Bars = 20 μm). **(C)** *In vivo* Co-IP assay of HA: MdAPX1 and GFP: VmPxE1 (without the signal peptide sequence). Both genes were expressed in *N. benthamiana* leaves using agro-infection. The input experiment was performed by western blot with the HA antibody and GFP antibody to confirm the expression of the two proteins. The mixed proteins were blended with GFP-trap agarose beads. The final eluent was analyzed by immunoblot using above-mentioned antibodies to detect VmPxE1 and MdAPX1. Asterisk represents target band.

**FIGURE 7:**
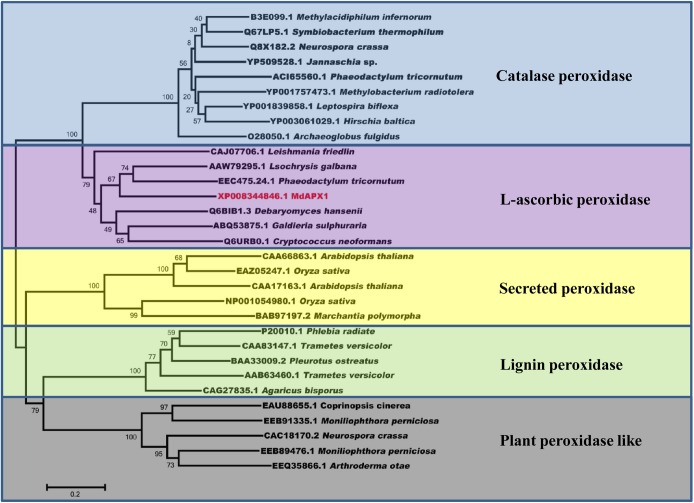
Sequence phylogenetic analysis of MdAPX1 showed that MdAPX1 was an L-ascorbic peroxidase, belonging to a subgroup of heme-dependent peroxidases of the plant superfamily. The unrooted phylogram was constructed based on the NJ method with 1000 bootstrap replicates.

To identify the target of VmPxE1, the GAL4 yeast two-hybrid system was performed using a *V. mali*-apple cDNA library. A potential gene segment was captured after screening tests on SD-Trp-Leu-His medium. The sequence was predicted as an apple ascorbate peroxidase protein, named as MdAPX1 (*Malus domestica* ascorbate peroxidase 1). Its intact coding sequence was constructed into the prey vector pGADT7. Auxotroph yeast strain AH109 was able to recover growth on SD-Trp-Leu-His when synchronously transformed with pGBKT7-*VmPxE1* and pGADT7-*MdAPX1* (**Figure 6A**). The dichotomous YFP segments were recovered and yellow fluorescence signal was detected in leaf cells of *N. benthamiana*, indicating that MdAPX1 could interact with VmPxE1 (**Figure 6B**). HA-MdAPX1 and GFP-VmPxE1 were transiently co-expressed in *N. benthamiana*, and total proteins went through GFP-trap gel beads for the specific adsorption. The immunoblotting showed that HA-MdAPX1 was present in the final GFP-VmPxE1-precipitated immunocomplex (**Figure 6C**), suggesting that MdAPX1 is a potential target of VmPxE1. Phylogenetic analysis showed that MdAPX1 (XP008344846.1) is an L-ascorbic peroxidase, belonging to a subgroup of heme-dependent peroxidases of the plant superfamily (**Figure 7**).

### Ectopic Expression of *MdAPX1*

**FIGURE 8:**
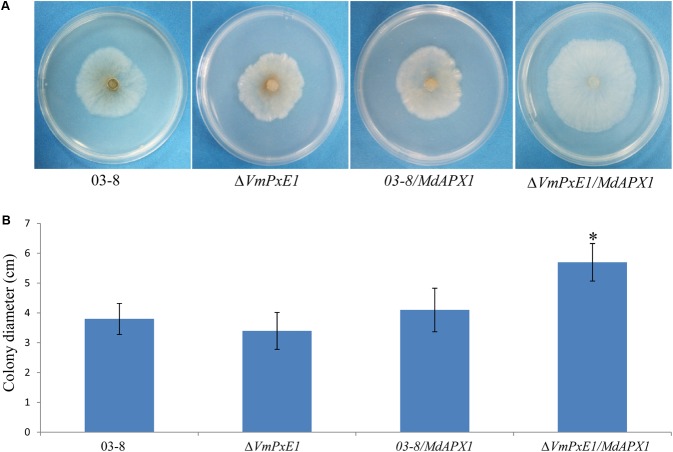
Ectopic expression of *MdAPX1* in *V. mali* wild type strain 03-8 and *VmPxE1* gene deletion mutant Δ*VmPxE1*. **(A)** Colony of strains on PDA supplemented with 0.06% H_2_O_2_, 3 dpi at 25°C. **(B)** Colony diameters. Bars indicate SD of the mean of 30 dishes and asterisk above the bar indicate significantly difference from 03-8 (*P* < 0.05).

The ectopic expression of *MdAPX1* in wild type 03-8 (*03-8/MdAPX1*) and *VmPxE1* gene deletion mutant strain (Δ*VmPxE1/MdAPX1*) was successful (**Supplementary Figure S3**). The growth of both the strains was significantly suppressed on PDA containing 0.06% H_2_O_2_. The transformed strain Δ*VmPxE1/MdAPX1* exhibited stronger resistance to H_2_O_2_ than the *03-8/MdAPX1* strain. These results suggested that *MdAPX1* enhanced the resistance of only Δ*VmPxE1* to H_2_O_2_ and that the presence of *VmPxE1* seems to have disturbed *MdAPX1* function (**Figure 8**).

## Discussion

We demonstrated that a cell death suppressor effector of *V. mali*, VmPxE1, was upregulated during the early stage of fungal infection of apple twigs and leaves, and identified one potential target of VmPxE1.

VmPxE1 from *V. mali*, a nectrophic pathogen, could suppress the BAX-induced PCD, which implies that VmPxE1 is involved in plant immune response, resulting in weakened host defense. This is supported by the fact that deletion of *VmPxE1* reduced virulence of *V. mali* but did not affect fungal vegetative growth and sporulation. Plant cell death induced by hypersensitive responses is believed to be detrimental for biotrophic pathogens, because of the reduction of vivosphere and restriction of hyphae extension (Gilchrist, 1998). On the other hand, plant cell death could facilitate the infection of necrotrophic pathogens and hence presence of effectors inducing cell death may be important for necrotrophic pathogens during the early infection process (Govrin and Levine, 2000; Wang et al., 2014). On the contrary, cell death suppressor effectors are vitally important for biotrophic and hemibiotrophic pathogens (Sharpee et al., 2017). However, the present study suggested that suppressing cell death may also play an important role in the infection by *V. mali*. Therefore, *V. mali* may not need to kill host cells rapidly but control cell death via these suppressors to enable successful colonization over time.

The target of VmPxE1 was identified to be an ascorbate peroxidase MdAPX1 from apple. Ascorbate peroxidase utilizes ascorbic acid as a specific electron donor and scavenges H_2_O_2_. During the plant–pathogen interaction, oxidative burst is concurrent and accompanied with the production of reactive oxygen species (ROS) (Apel and Hirt, 2004; Daudi et al., 2012). The ROS act as antimicrobial molecules and are also important as a signal related to plant disease resistance (Suzuki et al., 2011; Gilroy et al., 2014). However, excessive ROS would be phytotoxic. Plants possess a complex mechanism to balance the pernicious and beneficial effects of ROS (Daudi et al., 2012). Peroxidases act as an equalizer beam in the defense system of plant by using phenols, amine, or heterocyclic compound as hydrogen donor and preferring H_2_O_2_ as reaction substrates. Therefore, peroxidases could regulate H_2_O_2_ and concentrations of hydrogen donors (Welinder, 1992; Hiner et al., 2002; Yoshida et al., 2003; Camejo et al., 2016). Changes in H_2_O_2_ concentration could induce pathogenesis-related proteins and affect plant signals of disease resistance such as salicylic acid (Klessig and Malamy, 1994; Passardi et al., 2005), and is closely associated with hypersensitive cell death (Yoda et al., 2003). Apple tree possesses a large and complete peroxidase system that is well documented to be involved in its defense against pathogens (Camejo et al., 2016). The increased peroxidase activity in apple enhances resistance to Glomerella leaf spot (Araujo and Stadnik, 2013). Peroxidases increased activity associated with increased apple resistance to apple ring spot, blue mold, and apple scab (Valentines et al., 2005; Rachenko et al., 2014). These evidences suggest that this group of enzymes may play an important role in plant resistance to pathogens. Thus, it is not surprising that pathogens have to deal with peroxidases of its host. For instance *Ustilago maydis* effector Pep1 targets a maize peroxidase POX12 *in vivo* and suppresses the early immune responses of maize (Hemetsberger et al., 2012). Moreover, ascorbate peroxidase is reported to be closely related to plant resistance (Sarowar et al., 2005) and to act as a defense enzyme, enhancing systemic acquired resistance (Kvaratskhelia et al., 1997). We may, therefore, speculate that *MdAPX1* is involved in the apple defense system against pathogens. For successful infection, *MdAPX1* needs to be suppressed. Ectopic expression of *MdAPX1* in Δ*VmPxE1* (Δ*VmPxE1/MdAPX1*) showed stronger resistance to H_2_O_2_ than ectopic expression of *MdAPX1* in wild type 03-8 (*03-8/MdAPX1*), indicating that *VmPxE1* interfered the function of *MdAPX1*. Further research is needed to confirm whether and, if so, how *VmPxE1* disrupts the function of *MdAPX1*.

## Conclusion

We identified a cell death suppressor effector VmPxE1 from necrotrophic pathogen *V. mali* that contributes to fungal virulence and targets a peroxidase MdAPX1 of apple. Although, the putative effector in highly expressed during the apple infective process, *VmPxE1* was also expressed in basal conditions and an endogenous function could not be excluded. This data indirectly pointed that *VmPxE1* disturbs the function of MdAPX1. Further research is needed to confirm whether and, if so, how *VmPxE1* disrupts the function of *MdAPX1*.

## Author Contributions

LH, MZ, and HF contributed to the design of the work. MZ, HF, YZ, LS, and CG performed the experiments. MZ and YZ analyzed the sequencing data. MZ, HF, and XX wrote and revised the manuscript. LH was responsible for all aspects of this study.

## Conflict of Interest Statement

The authors declare that the research was conducted in the absence of any commercial or financial relationships that could be construed as a potential conflict of interest.

## Footnotes

1 http://pfam.xfam.org/

2 http://www.cbs.dtu.dk/services/SignalP/

## Supplementary Material

FIGURE S1 **(A)** RT-PCR detected *BAX*, *VmPxE1* and *eGFP* expression in *N. benthamiana* tissues at different locations: *VmPxE1* in location 1; *eGFP* in location 2; *VmPxE1* in location 3; *BAX* in location 3; *eGFP* in location 4; *BAX* in location 4; *GAPDH*. Total RNA was extracted 48 h after the second infiltration. **(B)** Validation of proteins expression of *BAX*, *HA: VmPxE1* by western blot with respective monoclonal murine antibodies injected in location 3.

FIGURE S2 Generation and identification of gene *VmPxE1* deletion and complementation mutants. **(A)** Flanks homologous recombination facilitated target gene replacement. The resistance gene cassette denoted by a gray arrow with homologous flank arms replaced the target gene denoted by a blue arrow based on homologous recombination. **(B)** Southern blot hybridization analysis of *VmPxE1* gene deletion mutants using primer NeoF/NeoR with digoxin marked nucleic fragment of *Neo* gene as hybridization probe. **(C)** Confirmation of *VmPxE1* knockout mutants by PCR analysis with four pairs of primers. The wild type 03-8 was as control. 1: 7F/Neo-CR detected upstream fusion segment. 2: Neo-CF/8R detected downstream fusion segment. 3: 5F/6R detected targeted gene and 4: Neo-CF/Neo-CR detected incoming resistant gene *Neo*. M: Maker. **(D)** PCR analysis was performed with primers 5F/6R to identify Δ*VmPxE1/VmPxE1* complementation mutant. 1: Wild type 03-8. 2: *VmPxE1* gene deletion mutants. 3: Δ*VmPxE1/VmPxE1* complementation mutant. M: Maker.

FIGURE S3 RT-PCR detects MdAPX1 and VmPxE1 in transformed strains *03-8/MdAPX1* and Δ*VmPxE1/MdAPX1* after cultured on PDA supplemented with 0.06% H_2_O_2_ 3 days at 25°C.

TABLE S1 Primers for cloning gene VmPxE1 to vectors, VmPxE1 gene deletion and PCR analysis in this study. The red color stands for corresponding directing sequences for homologous recombination and yellow color stands for corresponding restriction enzyme cutting site.

TABLE S5 primers for RT-PCR and qRT-PCR in this study.
