# Supplementary figures and images for: Valsa mali Pathogenic Effector VmPxE1 Contributes to Full Virulence and Interacts With the Host Peroxidase MdAPX1 as a Potential Target
Source: Front Microbiol. 2018 Apr 25;9:821. doi: 10.3389/fmicb.2018.00821 (PMC5996921; doi:10.3389/fmicb.2018.00821)

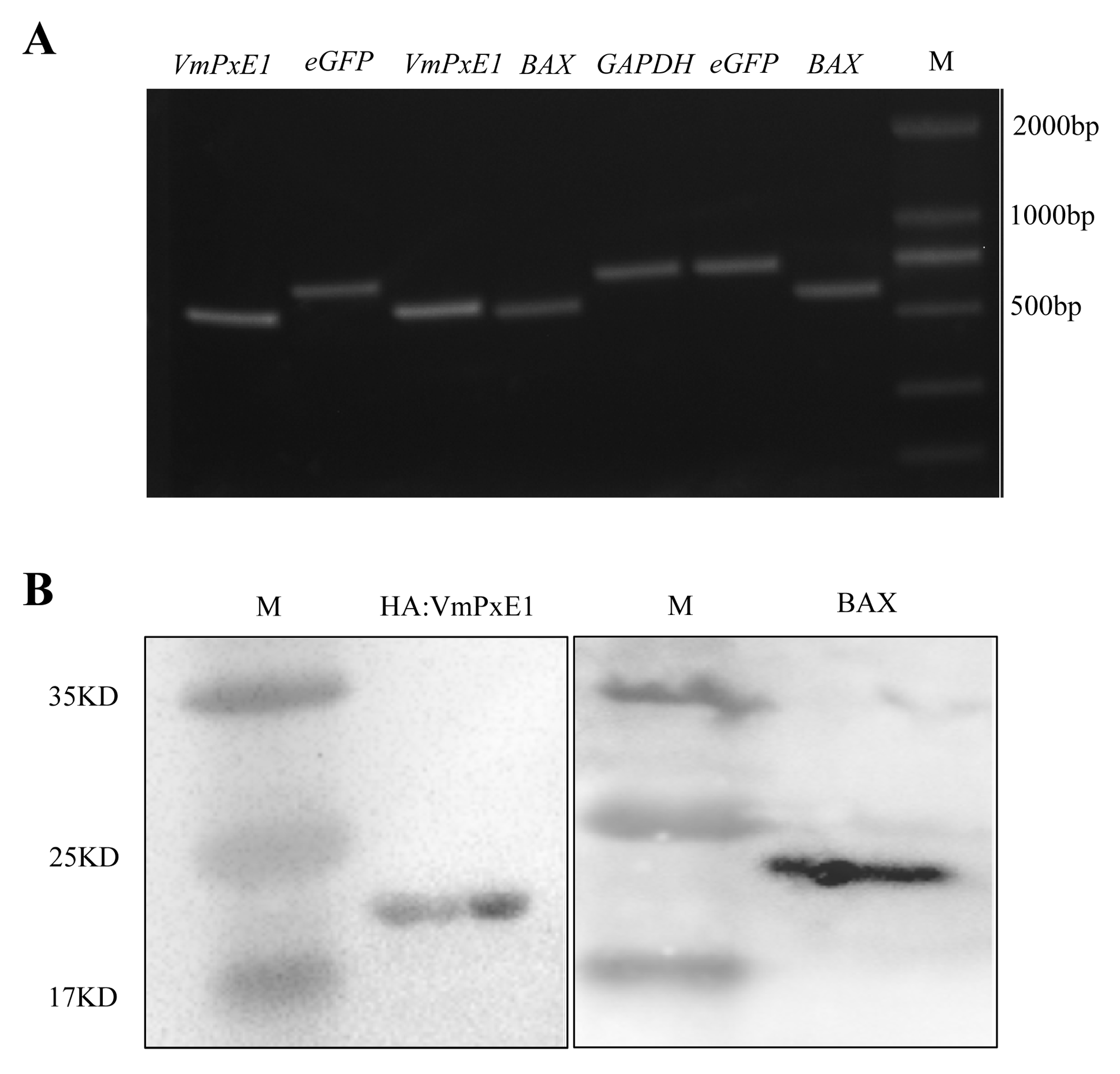

Supplement: FIGURE S1 — (A) RT-PCR detected BAX, VmPxE1 and eGFP expression in N. benthamiana tissues at different locations: VmPxE1 in location 1; eGFP in location 2; VmPxE1 in location 3; BAX in location 3; eGFP in location 4; BAX in location 4; GAPDH. Total RNA was extracted 48 h after the second infiltration. (B) Validation of proteins expression of BAX, HA: VmPxE1 by western blot with respective monoclonal murine antibodies injected in location 3. [file Image_1.TIF]

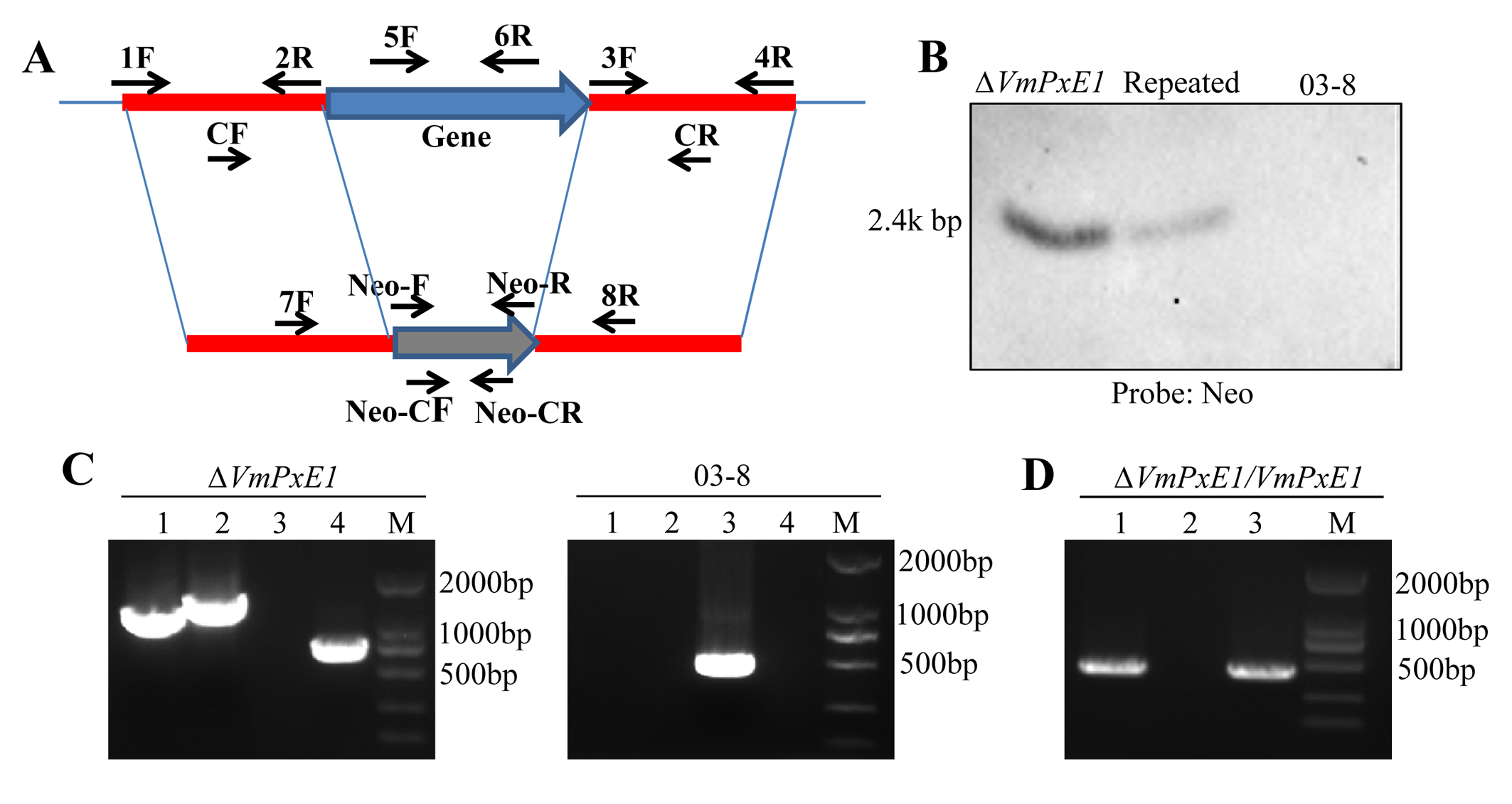

Supplement: FIGURE S2 — Generation and identification of gene VmPxE1 deletion and complementation mutants. (A) Flanks homologous recombination facilitated target gene replacement. The resistance gene cassette denoted by a gray arrow with homologous flank arms replaced the target gene denoted by a blue arrow based on homologous recombination. (B) Southern blot hybridization analysis of VmPxE1 gene deletion mutants using primer NeoF/NeoR with digoxin marked nucleic fragment of Neo gene as hybridization probe. (C) Confirmation of VmPxE1 knockout mutants by PCR analysis with four pairs of primers. The wild type 03-8 was as control. 1: 7F/Neo-CR detected upstream fusion segment. 2: Neo-CF/8R detected downstream fusion segment. 3: 5F/6R detected targeted gene and 4: Neo-CF/Neo-CR detected incoming resistant gene Neo. M: Maker. (D) PCR analysis was performed with primers 5F/6R to identify ΔVmPxE1/VmPxE1 complementation mutant. 1: Wild type 03-8. 2: VmPxE1 gene deletion mutants. 3: ΔVmPxE1/VmPxE1 complementation mutant. M: Maker. [file Image_2.TIF]

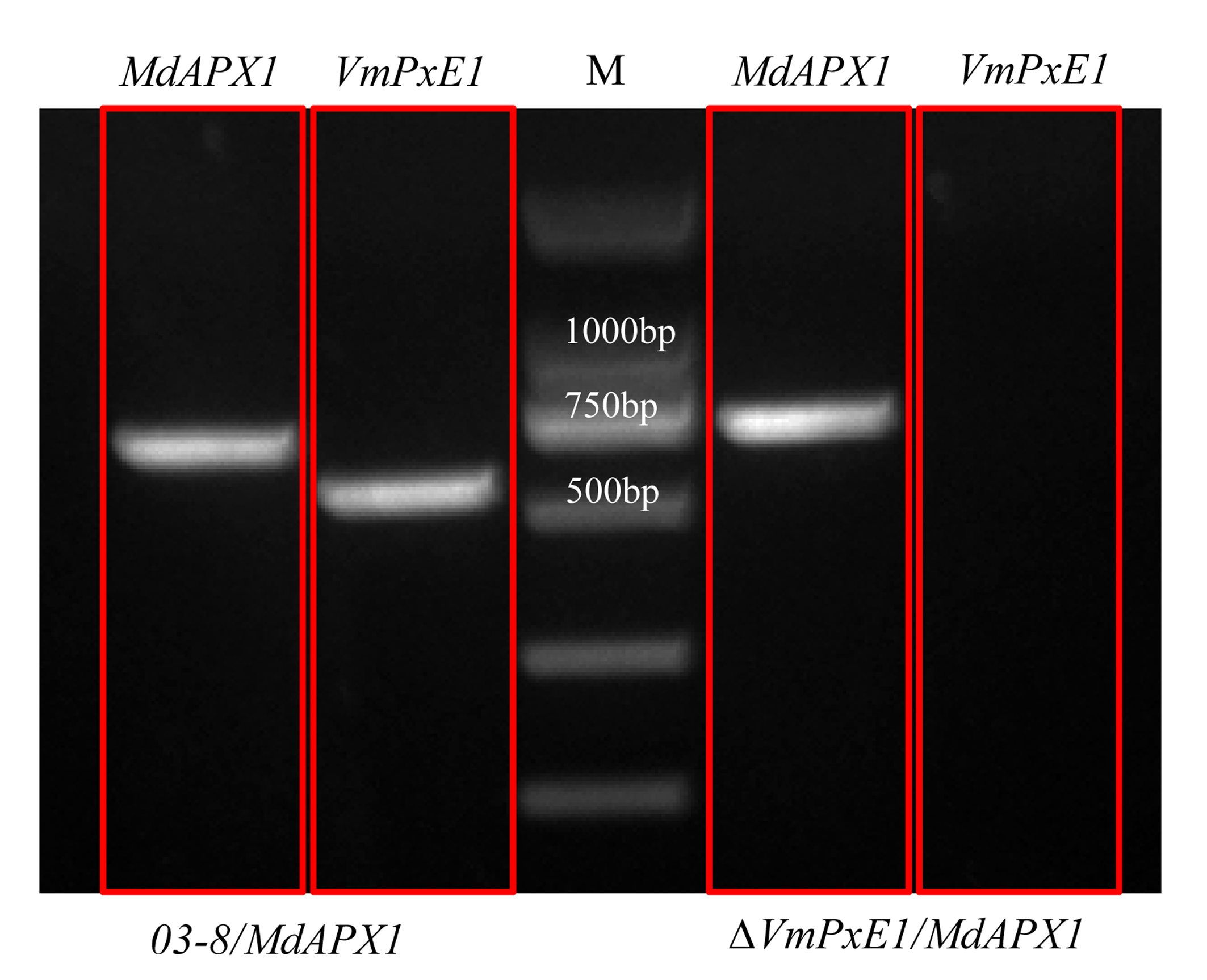

Supplement: FIGURE S3 — RT-PCR detects MdAPX1 and VmPxE1 in transformed strains 03-8/MdAPX1 and ΔVmPxE1/MdAPX1 after cultured on PDA supplemented with 0.06% H2O2 3 days at 25°C. [file Image_3.TIF]
