# Supplementary material for: Valsa mali Pathogenic Effector VmPxE1 Contributes to Full Virulence and Interacts With the Host Peroxidase MdAPX1 as a Potential Target
Source: Front Microbiol. 2018 Apr 25;9:821. doi: 10.3389/fmicb.2018.00821 (PMC5996921; doi:10.3389/fmicb.2018.00821)
Supplement: TABLE S1 — Primers for cloning gene VmPxE1 to vectors, VmPxE1 gene deletion and PCR analysis in this study. The red color stands for corresponding directing sequences for homologous recombination and yellow color stands for corresponding restriction enzyme cutting site. [file Table_1.DOCX]

Supplementary Material

*Valsa mali* pathogenic effector VmPxE1 contributes to full virulence and interacts with the host peroxidase MdAPX1 as a potential target

Mian Zhang^1^, Hao Feng^1^, Yuhuan Zhao^1^, Linlin Song^1^, Chen Gao^1^, Xiangming Xu^2^ and Lili Huang*^1^

^1^State Key Laboratory of Crop Stress Biology for Arid Areas, College of Plant Protection, Northwest A&F University, Taicheng Road 3, Yangling 712100, China

^2^NIAB East Malling Research, East Malling, Kent, ME19 6BJ, UK

*** Correspondence:** Lili Huang*

E-mail: [huanglili@nwsuaf.edu.cn](mailto:huanglili@nwsuaf.edu.cn)

| **Vectors** | **Sequence (5'- 3')** |
| --- | --- |
| PGR106-VmPxE1-F | CCATCGATATGAGGGCTCATATTCAATATCTAC |
| PGR106-VmPxE1-R | ACGCGTCGACTCACACCTTCACAGGCAACG |
| pSUC2-Vpsig-F | CGGAATTCATGAGGGCTCATATTCAATATCTAC |
| pSUC2-Vpsig-R | CCGCTCGAGCGCCGTAGCGAGTGACAC |
| pGBKT7-VmPxE1-F | ATGGCCATGGAGGCCGAATTCATGGACTGGTTCAGGCATGA |
| pGBKT7-VmPxE1-R | CCGCTGCAGGTCGACGGATCCTCACACCTTCACAGGCAACG |
| pGADT7-MdAPX-F | GGAATTCCATATGATGGGGAAGTGCTACCCTACC |
| pGADT7-MdAPX-R | CGGGATCCTTAGGCCTCAGCAAACCCAA |
| pSPYNE(R)173-VmPxE1-F | CGGGATCCATGGACTGGTTCAGGCATGA |
| pSPYNE(R)173-VmPxE1-R | GGGGTACCTCACACCTTCACAGGCAACG |
| pSPYCE(M)-MdAPX-F | GCTCTAGAATGGGGAAGTGCTACCCTACC |
| pSPYCE(M)-MdAPX-R | CGGGATCCGGCCTCAGCAAACCCAA |
| pBinGFP-VmPxE1-F | GGGGTACCATGGACTGGTTCAGGCATGA |
| pBinGFP-VmPxE1-R | CGGGATCCTCACACCTTCACAGGCAACG |
| PICH86998-MdAPX-F | TTACAATTATCGATACAATGATGTACCCATACGACGTCCCAGACTACGCTGGGAAGTGCTACCCTACC |
| PICH86998-MdAPX-R | CTCATTAAAGCAGGACAAGCTTAGGCCTCAGCAAACCCAA |
| 1F | GAGACTCAGTGAAGGGTGAGATTG |
| 2R | CAGATACGGCAGAGAAATCGCAACCTCCTTGCCGTCGAGACCAACA |
| 3F | GTTTAGATTCCAAGTGTCTACTGCTGGCATAGGAGAGGGAGGCTCCAGAA |
| 4R | CTCTCACAGAAGTAGCCAAACATCA |
| 5F | TGGACGCATTAGAAGACGC |
| 6R | CGATGATACAGGCACAACAGATA |
| CF | GGGTGAGATTGAAGAGATGTTCCT |
| CR | TGTGTCCTGACAGGTACACACTAAA |
| 7F | CGCCTCGTCTGAGCTCTAGAG |
| 8R | AGCGACTTGCAGGAGACGCT |
| Neo -F | GAGGTTGCGATTTCTCTGCCGT |
| Neo- R | GCCAGCAGTAGACACTTGGAATCT |
| Neo -CF | CAGCCCGATTTCCATTCCT |
| Neo- CR | CGGCGATACCGTAAAGCAC |
| PDL2-VmPxE1-F | CGACTCACTATAGGGCGAATTGGGTACTCAAATTGGTTATAATCCCTCCCCGACATGAGTAA |
| PDL2-VmPxE1-R | CCACCCCGGTGAACAGCTCCTCGCCCTTGCTCACCTCGAGCACCTTCACAGGCAACGCA |
| MdAPX1C-F | CATCGCCGAGAAGAACTGC |
| MdAPX1C-R | GTCCAAGGTCCTTCAAATCC |
| PDL2- MdAPX1 | CGACTCACTATAGGGCGAATTGGGTACTCAAATTGGTTATAAATGGGGAAGTGCTACCCTACC |
| PDL2- MdAPX1 | CCACCCCGGTGAACAGCTCCTCGCCCTTGCTCACCTCGAGTTAGGCCTCAGCAAACCCAA |

Table S1 Primers for cloning gene *VmPxE1* to vectors, *VmPxE1* gene deletion and PCR analysis in this study. The red color stands for corresponding directing sequences for homologous recombination and yellow color stands for corresponding restriction enzyme cutting site.

| **Gene** | **Primer for qRT-PCR/F** | **Primer for qRT-PCR/R** |
| --- | --- | --- |
| G6PDH (*Valsa mali*) | TCAGAACAAGTTCGAGGGCGACAA | TGAGGGCAATAGAGGGCTTGTTCA |
| VmPxE1 | GCCCTCACCCTCGGTATTG | GCTGCTTTGAACTGCTGGA |
| **Gene** | **Primer for RT-PCR/F** | **Primer for RT-PCR/R** |
| VmPxE1 | TGGCATCGAAGCAAGAAAC | GCAGTCACCAATGTCAACCC |
| GFP | ATGAGTAAAGGAGAAGAACTTTTCA | TTATTTGTATAGTTCATCCATGCCA |
| GAPDH (*Nicotiana benthamiana*) | CTCCACCCTTGGCATCTTT | GAGTGGTTCATCGCAGACAT |
| MdAPX1 | ATGGGGAAGTGCTACCCTACC | TTAGGCCTCAGCAAACCCAA |

Table S2 Primers for RT-PCR and qRT-PCR in this study.
